# Cell Surface Display of *Thermomyces lanuginosus* Lipase in *Pichia pastoris*

**DOI:** 10.3389/fbioe.2020.544058

**Published:** 2020-10-28

**Authors:** Jiaming Yang, Kui Huang, Xiaomin Xu, Yangli Miao, Ying Lin, Shuangyan Han

**Affiliations:** Guangdong Key Laboratory of Fermentation and Enzyme Engineering, School of Biology and Biological Engineering, South China University of Technology, Guangzhou, China

**Keywords:** *Thermomyces lanuginosus*, lipase, *Pichia pastoris*, cell surface display, whole cell biocatalyst

## Abstract

A cell surface displayed system in *Pichia pastoris* GS115 was developed by using GCW61, a glycosylphosphatidylinositol-modified cell wall protein from *P. pastoris*, as the anchor protein. *Thermomyces lanuginosus* lipase (*TL*L) was successfully displayed on the *P. pastoris* cell wall by fusing GCW61 gene with *TL*L2 gene (NCBI Accession: O59952) that was optimized with codon bias and synthesized. Cell surface displayed *TL*L2 was confirmed by the immunofluorescence microscopy. Flask fermentation was performed for 144 h with lipase activity up to 1964.76 U/g. Enzymatic properties of cell surface displayed *TL*L2 were also investigated. Displayed *TL*L2 occurred the maximum activity at pH 9 and 55°C and demonstrated characteristics of wide thermal adaptability and alkaline pH resistance. The optimum substrate was *p*-nitrophenyl hexanoate. Bivalent metal ions Ca^2+^, Mn^2+^, and Zn^2+^ had the activation effect on displayed *TL*L2, while Cu^2+^, Fe^2+^, Fe^3+^, K^+^, Li^+^, Na^+^, and Co^2+^ ions had the inhibitory effect on it. Since cell surface displayed *TL*L2 required less purification steps compared with free enzyme and showed high enzyme activities, it would be able to be further applied in various potential applications.

## Introduction

Lipases (Triacylglyceroyl hydrolase, EC 3.1.1.3) are one of the most extensive used enzymes in the biocatalyst, which can catalyze a wide range of biological transformations, including hydrolysis, esterification, interesterification, alcoholysis, acidolysis, and ammonolysis (Jaeger and Eggert, 2002; Yamada et al., 2016). Contrary to most enzymes, lipases exhibit a wide specificity, recognizing very different substrates. This permits them to be as catalysts for very different reactions in a wide range of applications, including detergent, food, medicine, fine chemicals, biodiesel, biosensors, and biodegradation (Rodrigues and Fernandez-Lafuente, 2010a,b). Among lipases, *Thermomyces lanuginosu* lipase (*TL*L) has attracted more and more attention due to its noticeable thermostability (Fernandez-Lafuente, 2010). *TLL* was immobilized on silicate and commercialized with the name of Lipozyme TLIM in 1994 as a detergent additive by Novozymes Co. Nowadays, as an promising industrial biocatalyst, *TL*L is able to be used in many different fields, e.g., detergents, cosmetics, modification of oils and fats, production and recycling of biodiesel, organic chemistry, etc. (Prathumpai et al., 2004; Fernandez-Lafuente, 2010; Huang et al., 2012; Li et al., 2014). However, limited by the complicated separation and purification steps, the high cost or price seemed to be the bottleneck for the industrial production.

Cell surface display enables proteins to be displayed on the microbial cell surface by fusion with anchoring components (Lee et al., 2003). Cell surface display of enzymes on bacteria or yeast has been found effective in bio-catalysis reaction like free enzymes and behave better resistance to organic solvents to some extent (Yuzbasheva et al., 2015). Cell surface display integrates expression and immobilization of the enzyme together thus there is no requirement of complicated purification process except fusing the interest protein with anchor proteins through the convenient genetic operation (Kondo and Ueda, 2004). It has been used in many fields, such as live vaccine development, library screening and bioconversion (Georgiou et al., 1997; Tateno et al., 2007; Qiao et al., 2019), and have become a powerful technique in recent years. Up to now, few reports on the yeast surface display of *TL*L have been published, except Dai et al. (2012).

As for the yeast surface display system, many kinds of cell wall proteins such as Sed1, GCW21, Pir1, Flo1, CWP2, and GCW 61 have been used as anchor proteins on the cell surface in recent years (Kondo and Ueda, 2004; Kojima et al., 2012; Zhang et al., 2013). Most of them are glycosylphosphatidylinositol-modified cell wall proteins (GPI-CWPs), which contains the N-terminal signal peptide and C-terminal GPI signal sequence. They are essential proteins for the maintenance of normal morphology in yeast cells, and firstly found in *Saccharomyces Cerevisiae* (Hamada et al., 1998). Zhang et al. (2013) discovered about 50 kinds of GPI-CWPs derived from *P. pastoris* and used CALB as the target protein displayed on the surface for experimental verification, of which 13 GPI-CWPs could be used as anchor proteins in the *P. pastoris* surface display system. Displayed CALB exhibited the highest enzyme activities when the anchor protein was substituted with GCW61(Zhang et al., 2013).

In order to achieve the high expression of surface display of *TL*L in *P. pastoris*, we established a new cell surface display system with GCW61 as the anchor protein. By fusing GCW61 gene with *TL*L2 gene optimized with codon bias, *TL*L2 was displayed on the outer of *P. pastoris* GS115 cell wall and demonstrated high hydrolytic activity. Then displayed *TL*L2 was evaluated in regard of the optimum temperature and pH, as well as the stability toward temperature, pH, and substrate. The achievement of cell surface displayed *TL*L2 with good performance would be benefit the production cost reduction of *TL*L and foundation for its large-scale industrial application.

## Materials and Methods

### Strains, Media and Growth Conditions

*Escherichia coli* TOP10F’ and *P. pastoris* GS115 (Invitrogen, United States) was used as a host strain for general cloning and a host strain for surface display studies, respectively. *E. coli* TOP10F’ was incubated at 37°C in Luria-Bertani medium (1% w/v tryptone, 0.5% w/v yeast extract, and 1% w/v NaCl) supplemented with 100 μg/mL kanamycin. *P. pastoris* was cultured at 30°C in following media: YPD (1% w/v yeast extract, 2% w/v peptone, and 2% w/v dextrose) for sub-cultivation, MD (1.34% w/v yeast nitrogen base, 2% w/v dextrose, 2% w/v agar) for the positive transformation selection, tributyrin plate (0.5% w/v ammonium sulfate, 0.3% w/v yeast extract, 0.5% w/v methanol, 2% w/v agar, 100 mM potassium phosphate pH 6, 0.5% w/v tributyrin) for the high expression strain selection, BMGY (1% w/v yeast extract, 2% w/v peptone, 100 mM potassium phosphate pH 6, 1.34% w/v yeast nitrogen base, and 1% v/v glycerol) for the cell growth, and BMMY (1% w/v yeast extract, 2% w/v peptone, 100 mM potassium phosphate pH 6, 1.34% w/v yeast nitrogen base, and 1% v/v methanol) for recombinant protein production.

### Construction of the Plasmid

*TL*L2 gene (NCBI Accession: O59952) from *Thermomyces lanuginosus* was synthesized (Generay, China) with an *Eco*RI restriction and a Flag-Tag at the 5′ end and *Mlu*I restriction site at 3′ end. *TL*L2 gene was amplified using primers *TL*L2-F (5′-ACCG GAATTCGAGGTTTCTCAGGATCTTTTTAACCAGTTCA-3′) and *TL*L2- R (5′-ATCGACGCGTCTTATCGTCGTCATCCTTG TAATCGAGGCAGGTCCCGATAAGTCCGA-3′). *TL*L2 gene and GCW61 gene (NCBI Accession: XM_002494287) are co-cloned into the expression plasmid pHKA (His4 + Kna + 3’ AOX gene segment in plasmid pPIC9K was used to replace the zeocin resistance gene in the pPICZα A using *Bam*HI and *Mlu*I sites), resulting in recombination plasmid pHKA-*TL*L2-GCW61. The plasmid was transformed into *E. coli* TOP10F’ competent cells and confirmed by using restriction enzyme digestion and DNA sequencing.

### Yeast Transformation

Plasmids were linearized using *Sal*I restriction enzyme (Takara, Japan) and transformed into *P. pastoris* GS115 competent cells via electroporation using the gene pulser apparatus (Bio-Rad, United States) with following parameters: 1500 V, 25 μF, and 200 V in a 0.2 cm cuvette. Electroporation was performed according to manufacturer’s instructions (Invitrogen, United States). Transformed cells were selected on MD agar plates, which had been incubated at 30°C for 2–3 days. Transformations were confirmed using the colony PCR, and the positive colony was transferred to the tributyrin plate for the high expression transformants screening.

### Cultivation of *P. pastoris* and Expression of the Lipase

Transformants harboring the plasmids for cell surface expression of *TL*L2 were inoculated into a 50 mL Erlenmeyer flask containing 10 mL of BMGY medium and pre-cultivated overnight at 30°C and 250 rpm. Next, primary culture was inoculated from pre-cultures to obtain the cell density of OD_600_ of 1. Cells were grown in 25 mL of BMMY medium in the 250 mL Erlenmeyer flask in a shaking incubator at 30°C and 250 rpm. Fresh methanol was added to obtain a final concentration of 2% v/v every 24 h. OD_600_ and lipase activity was monitored throughout a 7-days incubation.

### Immunofluorescence Microscopy

Immunofluorescence microscopy was performed according to the method reported previously (Kobori et al., 1992). *P. pastoris* was induced for 144 h in BMMY, and then were harvested by centrifugation for 1 min at 10,000 rpm and 4°C. The supernatant was discarded. Harvested cells were washed with distilled water and resuspended in ice-cold phosphate-buffered saline (PBS, pH 7.4), with 10 mg/mL of bovine serum albumin to block the cell surface. Anti-flag monoclonal antibody (Agilent, United States) was used as the primary antibody. Cell suspension was incubated with the primary antibody at a dilution of 1:200 in a total volume of 200 mL at room temperature for 2 h. Next, cells were washed twice with PBS and exposed to the secondary Alexa Fluor 488 goat anti-mouse IgG (H + L) antibody (Invitrogen, United States) at a final concentration of 10 ng/mL for 1 h at room temperature. Cells were washed three times with PBS and analyzed by using the BX51 fluorescence microscopy (Olympus, Japan). GS115/pPIC9K was also processed in the same procedure to serve as negative controls.

### Surface Displayed Lipase Hydrolytic Activity Assays

A modified lipase hydrolytic activity assay was used (Kobori et al., 1992). After induction with BMMY for expression of *TL*L2, the hydrolytic activity was assayed by measuring spectrophotometrically with *p*-nitrophenyl octanoate (Sigma, United States) as the substrate. *P*-nitrophenyl octanoate was emulsified by sonication in ultrapure water containing 0.5% w/v Triton X-100, resulting in a final concentration of 25 mM. One milliliter reaction solution, consisting of 900 μL of 50 mM Tris–HCl buffer (pH 9), 50 μL of cell suspension with appropriate dilution, and 50 μL *p*-nitrophenyl octanoate, was incubated for 5 min at 55°C. After the reaction was completed, the assay mixture was centrifuged at 10,000 rpm at room temperature for 1 min. Two-hundred microliter aliquot of the supernatant was added to a 96-well plate, and the absorbance was measured using a kinetic microplate reader (Molecular Devices, United States). Average values were generated from triplicates of each sample. The cell concentration was determined by the OD_600_ measurement. One unit of enzyme activity of cell surface displayed *TL*L2 was defined as the amount of enzyme that released l μmol *p-*nitrophenol (*p*NP) per minute from *p*-nitrophenyl octanoate under the assay conditions.

### Optimum Substrate

*P*-nitrophenol esters with different carbon chain lengths [*p-*nitrophenyl octanoate for *p-*nitrophenyl butyrate (Sigma, United States), *p-*nitrophenyl hexanoate (Ark Pharm, United States), *p-*nitrophenyl decanoate (Sigma, United States), *p-*nitrophenyl laurate (Sigma, United States), and *p-*nitrophenyl palmitate (Sigma, United States)] were used to determine the optimum substrate at a concentration of 25 mM for cell surface displayed *TL*L2. Operation procedures were the same as above, except replacing the substrate.

### Effect of pH and Temperature

The optimal pH for cell surface displayed *TL*L2 was determined by examining the hydrolytic activity at 55°C in the following buffer: 50 mM citric acid-sodium citrate buffer, pH 6; 50 mM Tris–HCl buffer, pH 7–9; 250 mM glycine-sodium hydroxide buffer, pH 10-11.

The optimum temperature for cell surface displayed *TL*L2 was determined by examining hydrolytic activity in 50 mM Tris–HCl buffer (pH 9), over the temperature range from 35 to 65°C.

To study the thermal stability, cell surface displayed *TL*L2 was incubated at 50, 60, 70, and 80°C for 90 min. The residual activity was measured every 15 min. The relative activity after incubation was determined by comparing the activity with that without thermal incubation.

To determine the pH stability, displayed *TL*L2 was incubated at 55°C for 3 h at different pH in the range of pH 7–11 (50 mM Tris–HCl buffer, pH 7–9; 50 mM glycine–NaOH buffer, pH 10–11), then the residual enzyme activity was measured.

The residual activity was measured by hydrolytic activity assays as described above. All measurements were done in triplicate. In Figures 5A,B,D, the maximum activity of cell surface displayed *TL*L2 under optimal conditions was taken as 100% and the relative activity of enzymes was defined as the ratio of enzyme residual activity to the maximum enzyme activity. In Figure 5C, the original activity before thermal treatment was taken as 100%.

### Effect of Metal Ions

Metal chloride solution [BaCl_2_, CaCl_2_, CoCl_2_, CuCl_2_, FeCl_2_, FeCl_3_, KCl, LiCl, MgCl_2_, MnCl_2_, NaCl, NiCl_2_, and ZnCl_2_ (concentration of 1, 5, and 10 mM)] was used to determine the effect of metal ions on the lipase activity. Cell surface displayed *TL*L2 was incubated in metal chloride solution and the residual activity was measured.

## Results and Discussion

### Construction of the *P. pastoris* Surface Display System

**FIGURE 1:**
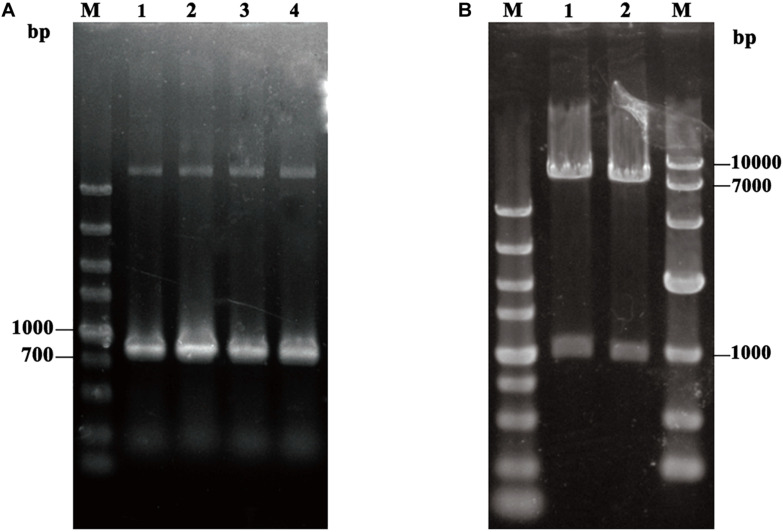
**(A)** PCR amplification of *TL*L2. M: DL5000 DNA Marker; Line1–Line4: the product of *TL*L2 by PCR. **(B)** Identification of the recombinant plasmid pHKA-*TL*L2-GCW61. M(Left): DL5000 DNA Marker; M(Right): DL10000 DNA Marker; Line1 and Line2: the product of pHKA-*TL*L2-GCW61 by double digests.

In order to display *TL*L on the surface of *P. pastoris* GS115, approximate 900 bp fragment of *TL*L2 gene was synthesized and amplified (Figure 1A), then ligated with an about 160 bp fragment of anchor glycoprotein GCW61 gene into the pHKA expression vector, resulting in recombinant plasmid pHKA-*TL*L2-GCW61. Plasmids were confirmed by double restriction enzyme digestion (Figure 1B) and DNA sequencing.

**FIGURE 2:**
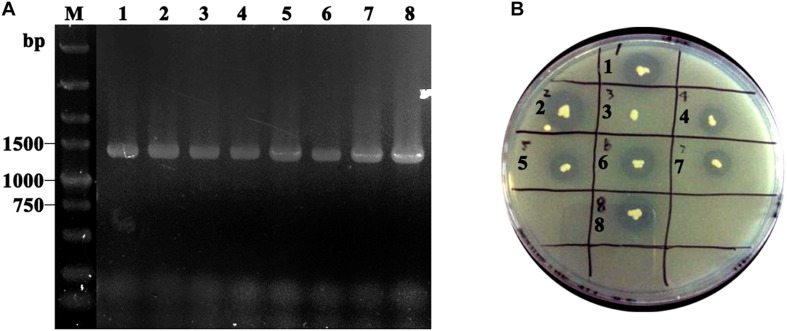
**(A)** Identification of GS115/pHKA-*TL*L2-GCW61 by colony PCR. M: DL5000 DNA Marker; line1–8: the colony PCR of GS115/pHKA-*TL*L2-GCW61. **(B)** Halo formation of GS115/pHKA-*TL*L2-GCW61 on the tributyrin plate.

Obtained plasmids were linearized using *Sal*I and transformed into *P. pastoris* GS115 competent cells. After MD plate screening, positive transformants were verified using colony PCR with *TL*L2-F and 3’AOXI primer pair (Figure 2A). To select the high expression colony, positive transformants were further transferred on tributyrin plate (Figure 2B). The colony GS115/pHKA-*TL*L2-GCW61#8 with bigger hydrolytic circle was picked to be stored and conducted fermentation by flask.

**FIGURE 3:**
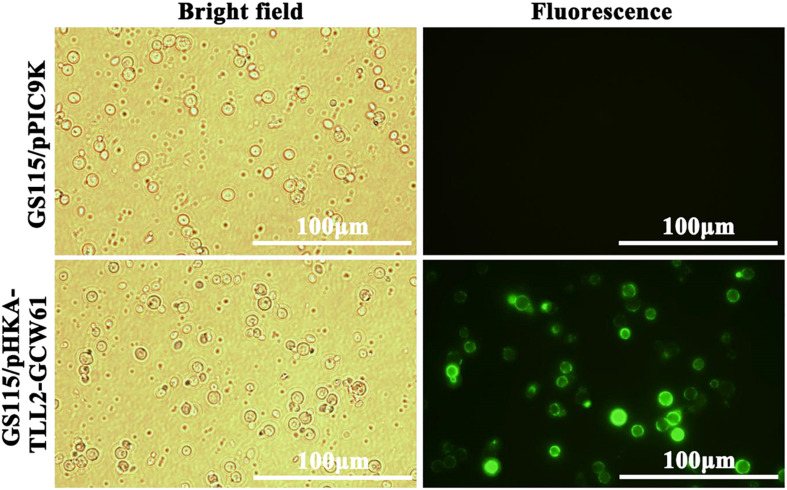
Detection of the recombinant *P. pastoris* with the immunofluorescence microscopy.

After 168 h of induction with methanol, cells were harvested, and immunofluorescence microscopy of cells was performed. The green fluorescence of the immunostaining GCW61 fusion protein was clearly observed outlining *P. pastoris* GS115 harboring recombinant plasmid of pHKA-*TL*L2-GCW61. In contrast, little fluorescence was emitted by the control strain GS115/pPIC9K (Figure 3). This confirmed that *TL*L2-GCW61 fusion proteins were anchored on the *P. pastoris* GS115 surface.

### Productivity of the *P. pastoris* Surface Display System

**FIGURE 4:**
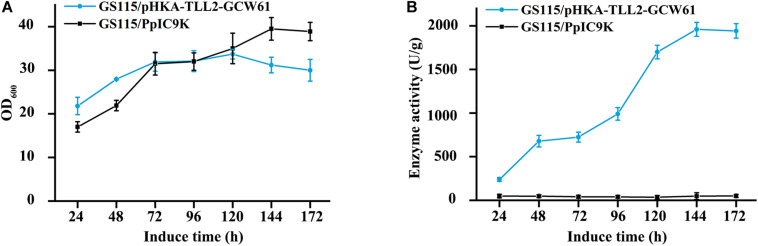
**(A)** Growth curve of GS115/pHKA-*TL*L2-GCW61. **(B)** Hydrolysis activity of GS115/pHKA-*TL*L2-GCW61.

GS115/pHKA-*TL*L2-GCW61#8 was selected to be fermented in flask, and GS115/pPIC9K was used as the negative control. From the growth curve of GS115/pHKA-*TL*L2-GCW61#8 (Figure 4A), the growth rate of surface displayed cells began to reach a plateau after 72 h of induction, which showed heterologous protein expression might have a certain adverse impact on the growth of host cells in the induced expression period. However, lipase activity retained increasing after 72 h and doubled within the following 48 h (72–120 h). After 144 h induction expression of transformants GS115/pHKA-*TL*L2-GCW61#8, the lipase hydrolysis activity reached 1964.76 U/g (Figure 4B).

**FIGURE 5:**
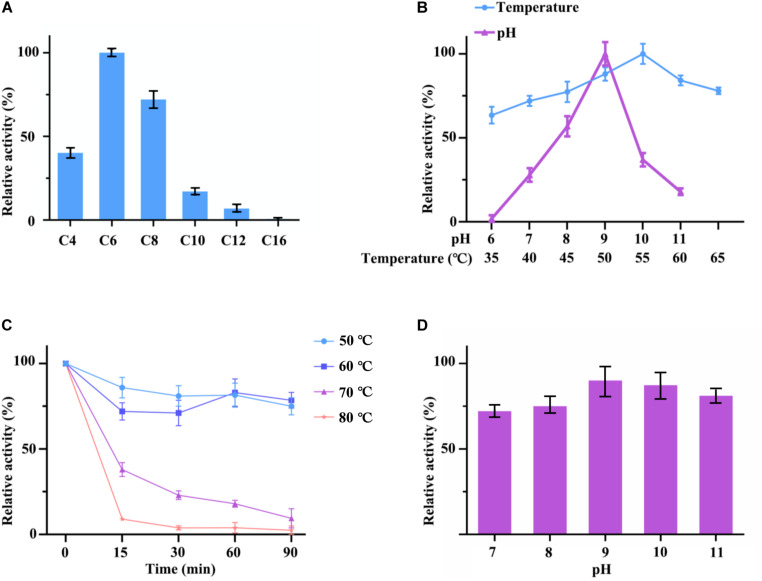
**(A)** Optimum substrate of hydrolysis activity of cell surface displayed *TL*L2. C4 stands for *p*-nitrophenyl butyrate; C6 stands for *p*-nitrophenyl hexanoate; C8 stands for *p*-nitrophenyl octanoate; C10 stands for *p*-nitrophenyl decanoate; C12 stands for *p*-nitrophenyl laurate; C16 stands for *p*-nitrophenyl palmitate. **(B)** Optimum pH and temperature of cell surface displayed *TL*L2. **(C)** Thermal stability of cell surface displayed *TL*L2. **(D)** pH stability of cell surface displayed *TL*L2.

### Enzymatic Properties of Cell Surface Displayed *TL*L2

The lipase activity of cell surface displayed *TL*L2 were detected by using *p*-nitrophenol esters with different carbon chain lengths as substrates. The results showed that hydrolytic activities of C4 (*p*-nitrophenyl butyrate), C6 (*p*-nitrophenyl hexanoate), and C8 (*p*-nitrophenyl octanoate) were higher than other *p*-nitrophenyl esters above C10, which suggested that cell surface displayed *TL*L2 preferred to catalyze the short and medium length chain of aliphatic acid ester. The optimum substrate was *p*-nitrophenyl hexanoate (Figure 5A).

As for pH and temperature, cell surface displayed *TL*L2 exhibited satisfactory alkaline pH and thermal adaptability (Figure 5B). The optimum pH was approximate pH 9, which is similar to the free *TL*L (NCBI Accession: AF054513) previously reported by Zheng et al. (2011). The maximum activity of cell surface displayed *TL*L2 occurred at 55°C, which is much higher than the optimum temperature of 30°C for the cell surface displayed *TL*L (NCBI Accession: AF054513) using Sed I as the anchor protein (Dai et al., 2012). In addition, cell surface displayed *TL*L2 showed above 70% activity from 40 to 65°C.

Moreover, when we focused the pH and thermal stability, the displayed *TL*L2 showed pretty good performance. As shown in the Figure 5C, cell surface displayed *TL*L2 retained approximately 70% of the original activity when it was incubated at 60°C for 90 min, which is almost coincides with the curve of 50°C. As for pH, more than 70% of lipase activities still retained after being preserved in pH 7 to pH 11 for 3 h that demonstrated the displayed *TL*L2 had good pH tolerance (Figure 5D).

Tables 1–3 showed the effect of metal ions on displayed *TL*L2 enzyme activity. As shown in Table 1, divalent metal ions Ca^2+^, Mn^2+^, and Zn^2+^ strongly activated the cell surface displayed *TL*L2. As for Zn^2+^, it was different from the results reported by Zheng et al. that Zn^2+^ has a certain inhibitory effect on free *TL*L (Zheng et al., 2011). There is a possible reason that the *TL*L2 spatial structure was influenced by the fusion protein GCW61, resulting in a change of dependence on some metal ions. Calcium ion was the strongest activator, and the *TL*L2 activity was found to be increased up to 839.36% when the calcium is at the concentration of 10mM. Another two groups, Cu^2+^, Fe^2+^, Fe^3+^, K^+^, Li^+^, Na^+^, Co^2+^ (Table 2) and Ba^2+^, Mg^2+^, Ni^2+^ (Table 3) had little activated and inhibitory effect on cell surface displayed *TL*L2, respectively.

**TABLE 1:** Metal ions with activation on cell surface displayed *TL*L2.

Metal ions	1 mM (%)	5 mM (%)	10 mM (%)
None	100.00	100.00	100.00
Ca^2+^	735.36	789.29	839.36
Mn^2+^	338.58	615.88	568.80
Zn^2+^	765.47	897.53	636.78

**TABLE 2:** Metal ions with inhibition on cell surface displayed *TL*L2.

Metal ions	1 mM (%)	5 mM (%)	10 mM (%)
None	100.00	100.00	100.00
Co^2+^	66.59	78.43	126.44
Cu^2+^	39.18	15.51	59.63
Fe^2+^	28.16	39.70	53.63
Fe^3+^	79.78	41.35	46.97
K^+^	74.08	54.38	25.24
Li^+^	59.78	56.10	28.84
Na^+^	61.87	58.65	18.73

**TABLE 3:** Metal ions with insignificant effect on cell surface displayed *TL*L2.

Metal ions	1 mM (%)	5 mM (%)	10 mM (%)
None	100.00	100.00	100.00
Ba^2+^	118.13	212.36	189.44
Mg^2+^	90.94	103.45	94.01
Ni^2+^	160.00	199.70	142.32

## Conclusion

*Thermomyces lanuginosus* lipase has broad application prospects due to its thermostability and substrate specificity. However, the low expression level of *TL*L and complexity of separation and purification process raises cost of the industrialized application. For this reason, the development and utilization of cell surface display to acquire higher performance *TL*L catalyst become the promising alternative way. This study reported the construction, expression and characterization of cell surface displayed *TL*L2 using GCW61 as the anchor protein in *Pichia pastoris*. Displayed *TL*L2 was relatively stable from pH 7 to 11 and retained approximately 70% of the original activity at 60°C for 90 min. Its maximal activities were observed at 55°C and pH 9. The high hydrolytic activity of 1964.76 U/g was obtained when the engineered strain was performed with flask methanol induced fermentation for 144 h. Displayed *TL*L2 exhibited higher activities in most kinds of metal ion solution, and could be strengthened by some kinds of metal ions, such as Ca^2+^, Mn^2+^, and Zn^2+^. Besides, displayed *TL*L2 preferred for short and medium length chain of aliphatic acid ester as substrates. These results indicate that cell surface displayed *TL*L2 might be suitable for a new cost-saving substitute for immobilized *TL*L in industry.

## Data Availability Statement

The datasets presented in this study can be found in online repositories. The names of the repository/repositories and accession number(s) can be found in the article/supplementary material.

## Author Contributions

JY: experimental verification, data curation, writing–reviewing and editing. KH: experiment performing and draft preparation. XX: experimental assistance and software. YM: methodology. YL: supervision. SH: conceptualization and funding acquisition. All authors contributed to the background research and writing of the article, as well as the editing. In addition, all authors have read and approved the final version of this manuscript.

## Conflict of Interest

The authors declare that the research was conducted in the absence of any commercial or financial relationships that could be construed as a potential conflict of interest.
